# Molecular Mechanisms Underlying Chronic and Degenerative Diseases

**DOI:** 10.3390/ijms241512507

**Published:** 2023-08-07

**Authors:** Alessandro Cannavo

**Affiliations:** Department of Translational Medical Sciences, University of Naples Federico II, 80131 Naples, Italy; alessandro.cannavo@unina.it

The Special Issue entitled “Molecular Mechanisms Underlying Chronic and Degenerative Diseases” contains eight articles: six original studies and two reviews. The need for such a Special Issue is related to the increasingly emerging concern about chronic degenerative disorders, which have become a growing pandemic for older adults. Although there is a greater comprehension of the mechanisms underlying the pathogenesis of these disorders today, current therapies still have limited impact, and mortality remains too high. Thus, there is a keen interest in comprehending the mechanisms behind the pathogenesis of chronic degenerative disorders, supplying the basis for identifying novel therapeutic targets and generating new treatments. 

Considering this premise, the first purpose of this Special Issue is to provide an update on the most advanced knowledge and comprehensive elucidation of molecular mechanisms involved in such disorders. 

To this aim, Zhai and coworkers’ original research [1] article explores the role of cardiomyocyte metabolism in heart failure (HF) pathogenesis. HF is a chronic progressive disease representing a global health problem since it involves millions of people worldwide [2,3]. Despite the progress in the field, the prevalence of HF is dramatically increasing, calling for a deeper understanding of specific mechanisms and pathological perturbations that pave the way for the development and progression of this disorder. The G-protein-coupled receptor (GPCR) kinase-2 (GRK2) is critically involved in the development and progression of HF [3]. Indeed, the elevated levels and activity of GRK2 promote the dysfunction of cardiac and adrenal α- and β-ARs and regulate cardiomyocyte death and metabolism via its mitochondrial localization and signaling [3]. The study by Zhai et al. [1] further supports the importance of GRK2 inhibition or its genetic deletion as a potent therapeutic strategy in HF. Indeed, these authors provide evidence that increased cardiac GRK2 levels, even without cardiac injury/stress, can modify cardiomyocytes’ bioenergetic homeostasis, contributing to cardiomyocyte death and HF progression.

For their part, Ruz et al. [4], in their original research article, analyze the mechanisms involved in the pathogenesis of Parkinson’s disease (PD). PD is an age-related chronic neurodegenerative disorder, and decades of research, among other risk factors, have established the importance of rare and common genetic variants. In this regard, these authors explore the relationship between leucine-rich repeat kinase 2 (LRRK2) and the *GBA1 *gene, which encodes for glucocerebrosidase 1 (GCase), a lysosomal enzyme that catalyzes the hydrolysis of glucosylceramide to glucose and ceramide [5]. Notably, mutations in *LRRK2* or *GBA1* genes or alterations in their enzymatic activities are well-recognized contributors to PD pathogenesis, but despite this, the specific mechanistic interplay between these two molecules remains undetermined. In this sense, Ruz and colleagues [4] reported that the inhibition of LRRK2 kinase activity in a human neuroglioma cell model resulted in increased GBA protein levels with no changes in the overall GCase activity compared to the control cells. This study suggests a possible regulation of lysosomal function through LRRK2 and supports an interplay between LRRK2 kinase activity and GBA.

Significantly, mutations in LRRK2 may also affect the expression of Synaptogyrin-3 (SYNGR3), a synaptic-vesicle-associated protein whose reduced expression has been observed in Parkinson’s disease (PD) in both humans and rodents [6,7]. Of note, the specific mechanism responsible for regulating SYNGR3 expression (at genomic or protein levels) in neurons was investigated by Li et al. [7]. In detail, these authors conducted an in silico investigation of nucleotide sequence spanning both the 5′-flanking region and 5′-UTR of SYNGR3. Through this approach, they identified three putative non-canonical nerve-growth-factor-induced clone B (NGFI*-*B) response element (ncNBRE) sites in the 5′-flanking region of SYNGR3 that bind the nuclear-receptor-related-1 protein (NURR1), a nuclear receptor superfamily member whose role has been described with regard to the etiology of PD. Interestingly, the authors demonstrated that the NURR1-dependent regulation of SYNGR3 expression is neuron specific, and therefore proposed NURR1 activators as a potential strategy to enhance SYNGR3 expression and attenuate synaptic dysfunction in PD.

The second objective of this Special Issue is to report the most recent clinical evidence linking molecular mechanisms with the pathogenesis of chronic degenerative diseases.

In line with this purpose, the review article by Ehtewish and colleagues [8] provides an exhaustive background on the potential risk factors and the underlying mechanisms linking diabetes, cognitive impairment, and dementia. Moreover, these authors discuss the most relevant biomarkers related to cognitive decline in patients with diabetes. Notably, the identification and usage of biomarkers in basic and clinical research and practice is now accepted without question. Based on their definition, biomarkers are measurable and evaluable indicators that help to define physiological and pathological processes, and their usefulness is essential to monitor the response to specific therapeutics. In this context, the review article by Hermenean et al. [9] discusses the role of Galectins, particularly Galectin-1, as a biomarker for the diagnosis and prognosis of tissue fibrosis, along with its therapeutical inhibition, in different chronic diseases.

Next, in their original article, Mayeli and colleagues [10] contribute to this Special Issue with a preliminary study that aims to identify novel potential molecular pathophysiological biomarkers for chronic and deteriorating mental illnesses such as schizophrenia. Growing evidence suggests that excitatory and inhibitory (E/I) activity alterations characterize the dorsolateral prefrontal cortex (DLPFC) of subjects with schizophrenia. Notably, the E/I balance is essential for efficient neuronal activity in the brain, and it is maintained by a functional balance between the expression and activity of the glutamate (Glu)-ergic (excitatory) and Gamma-aminobutyric acid (GABA)-ergic (inhibitory) systems. Therefore, in this report, these authors utilized 7-Tesla magnetic resonance spectroscopic imaging (MRSI) to measure Glu, GABA, and Glu/GABA in both left and right DLPFC in subjects with first-episode schizophrenia, a clinical high risk, and healthy comparison. These authors demonstrated that GABA and Glu/GABA in the DLPFC may represent a putative early pathophysiological biomarker for schizophrenia and other related disorders. 

For their part, Folke and coworkers [11] evaluated high-affinity anti-alpha-synuclein (αSyn) naturally occurring autoantibodies (nAbs) in blood sera from patients with PD and multiple-system atrophy (MSA), which are neurodegenerative disorders characterized by the pathological deposition of aggregated αSyn. Interestingly, these authors reported aberrancies in the anti-αSyn nAb compartment in the MSA and PD prodromal stages, providing novel insights into the possible causes behind the pathogenesis of these disorders.

Finally, in their study, Wang et al. [12] analyzed the effects of Simvastatin (SV) in the treatment of benign prostatic hyperplasia (BPH), a common condition in men aged ≥50 years. Notably, a relationship between metabolic syndrome (MetS) and BPH was observed [13,14,15], and SV, an established statin for the treatment of MetS, was proven to induce beneficial effects in patients with BPH [16]. Significantly, these authors demonstrated that the SV treatment of human prostate cells (BPH-1 and WPMY-1) in vitro, and of rats in vivo, resulted in an augmented cell death rate and attenuated fibrosis and epithelial–mesenchymal transition (EMT) process. Mechanistically, these effects were attributed to an SV-mediated upregulation of the peroxisome-proliferator-activated receptor gamma (PPARγ), which, in turn, inhibited WNT/β-catenin signaling. 

Chronic degenerative disorders such as HF and neurodegenerative disorders are a growing pandemic for older adults. Currently, no available treatment can slow the progression or incidence of these diseases, with massive healthcare costs predicted to rise constantly in the coming years. 

Overall, the exciting contributions of this Special Issue expand our knowledge about some of the mechanisms involved in the pathogenesis of such disorders, providing a basis for identifying and developing druggable targets and biomarkers to be implemented in clinical practice and offering benefits to patients who are affected by or at risk for developing these progressive, chronic, and disabling diseases.
